# Artificial Intelligence Versus Clinicians in Disease Diagnosis: Systematic Review

**DOI:** 10.2196/10010

**Published:** 2019-08-16

**Authors:** Jiayi Shen, Casper J P Zhang, Bangsheng Jiang, Jiebin Chen, Jian Song, Zherui Liu, Zonglin He, Sum Yi Wong, Po-Han Fang, Wai-Kit Ming

**Affiliations:** 1 Department of Obstetrics and Gynecology The First Affiliated Hospital of Sun Yat-sen University Guangzhou China; 2 School of Medicine Jinan University Guangzhou China; 3 School of Public Health The University of Hong Kong Hong Kong China (Hong Kong); 4 International School Jinan University Guangzhou China; 5 Faculty of Medicine Jinan University Guangzhou China; 6 College of Information Science and Technology Jinan University Guangzhou China; 7 School of International Studies Sun Yat-sen University Guangzhou China; 8 Harvard Medical School Harvard University Boston, MA United States; 9 Division of Pharmacoepidemiology and Pharmacoeconomics Brigham and Women’s Hospital Boston, MA United States

**Keywords:** artificial intelligence, deep learning, diagnosis, diagnostic imaging, image interpretation, computer-assisted, patient-centered care

## Abstract

**Background:**

Artificial intelligence (AI) has been extensively used in a range of medical fields to promote therapeutic development. The development of diverse AI techniques has also contributed to early detections, disease diagnoses, and referral management. However, concerns about the value of advanced AI in disease diagnosis have been raised by health care professionals, medical service providers, and health policy decision makers.

**Objective:**

This review aimed to systematically examine the literature, in particular, focusing on the performance comparison between advanced AI and human clinicians to provide an up-to-date summary regarding the extent of the application of AI to disease diagnoses. By doing so, this review discussed the relationship between the current advanced AI development and clinicians with respect to disease diagnosis and thus therapeutic development in the long run.

**Methods:**

We systematically searched articles published between January 2000 and March 2019 following the Preferred Reporting Items for Systematic reviews and Meta-Analysis in the following databases: Scopus, PubMed, CINAHL, Web of Science, and the Cochrane Library. According to the preset inclusion and exclusion criteria, only articles comparing the medical performance between advanced AI and human experts were considered.

**Results:**

A total of 9 articles were identified. A convolutional neural network was the commonly applied advanced AI technology. Owing to the variation in medical fields, there is a distinction between individual studies in terms of classification, labeling, training process, dataset size, and algorithm validation of AI. Performance indices reported in articles included diagnostic accuracy, weighted errors, false-positive rate, sensitivity, specificity, and the area under the receiver operating characteristic curve. The results showed that the performance of AI was at par with that of clinicians and exceeded that of clinicians with less experience.

**Conclusions:**

Current AI development has a diagnostic performance that is comparable with medical experts, especially in image recognition-related fields. Further studies can be extended to other types of medical imaging such as magnetic resonance imaging and other medical practices unrelated to images. With the continued development of AI-assisted technologies, the clinical implications underpinned by clinicians’ experience and guided by patient-centered health care principle should be constantly considered in future AI-related and other technology-based medical research.

## Introduction

### Background

An aging patient population and a shortage of medical professionals have led to a worldwide focus on improving the efficiency of clinical services via information technology. Artificial intelligence (AI) is a field of algorithm-based applications that can simulate humans’ mental processes and intellectual activity and enable machines to solve problems with knowledge. In the information age, AI is widely used in the medical field and can promote therapeutic development. AI may optimize the care trajectory of patients with chronic disease, suggest precision therapies for complex illnesses, and reduce medical errors [1].

There are currently 2 common types of AI. The first type is expert systems. An expert system is a computer system that generates predictions under supervision and can outperform human experts in decision making. It consists of 2 interdependent subsystems: a knowledge base and an inference engine. Although the knowledge base contains accumulated experience, the inference engine (a reasoning system) can access the current state of the knowledge base and supplement it with new knowledge. Expert systems can create more explicit critical information for the system, make maintenance easy, and increase the speed of prototyping [2]. However, expert systems are limited regarding knowledge acquisition and performance. Computer-assisted techniques have been introduced in medical practice for decades but have recently yielded minimal improvements. The second type is machine learning. This is the core of AI and is a fundamental approach to making computers intelligent. Machine learning requires vast amounts of data for training. This systematically improves their performance during the process. One of the focuses underlying machine learning is parameter screening. Too many parameters can lead to inaccurate entries and calculations; therefore, reducing the number of parameters can improve the efficiency of AI, but it may also lower its accuracy. However, 1 of the critical objectives of AI is to outperform humans via self-study in challenging fields without any previous knowledge.

AI has been extensively used in a range of medical fields. Clinical diagnoses of acute and chronic diseases, such as acute appendicitis [3] and Alzheimer disease [4], have been assisted via AI technologies (eg, support vector machines, classification trees, and artificial neural networks). Integrative AI consisting of multiple algorithms rather than a single algorithm substantially improves its abilities to detect malignant cells, yielding higher diagnostic accuracy [5].

The development of diverse AI techniques also contributes to the prediction of breast cancer recurrence [6]. In-home AI systems may potentially oversee patients with insulin abnormalities and swallowing problems [7] rather than doctors. Treatment optimization is achievable by AI [8] for patients with common, but complex diseases characterized as being ascribed to multiple factors (eg, genetic environmental or behavioral) such as cardiovascular diseases are more likely to benefit from more precise treatments on account of the AI algorithms based on big data [8]. On the other hand, AI-assisted hospital management systems could also help minimize logistics-associated monetary and temporal costs on a larger scale [9].

### Objectives

To our knowledge, there is no published review comparing the diagnostic performance between AI and clinicians. Thus, we aimed to systematically review the literature and provide an up-to-date summary indicating the extent of application of AI to disease diagnoses compared with clinicians. We hope this review would help foster health care professionals’ awareness and comprehension of AI-related clinical practices.

## Methods

### Search Strategy, Selection Criteria, and Study Selection

This search strategy was developed upon consultation with a professional librarian. The literature search was conducted in Scopus (the largest abstract and citation database spanning multiple disciplines), PubMed, CINAHL, Web of Science, and Cochrane Library using the combination of searching terms (see Multimedia Appendix 1). The search was limited to articles published between January 2000 and March 2019 following the Preferred Reporting Items for Systematic reviews and Meta-Analysis. Additional potentially eligible articles were manually searched via screening of the reference list of included articles as well as our personal archives.

We included articles if they (1) focused on advanced AI (defined as an AI encompassing a training or *learning* process to automate expert-comparable sophisticated tasks), (2) enclosed at least an application to particular disease diagnoses, (3) compared the performance between AI and human experts on specific clinical tasks, and (4) were written in English. Articles were excluded if they (1) only described simpler AIs that do not involve any training or *learning* process; (2) did not compare performance of AI with that of medical experts; and (3) were conference abstracts, book chapters, reviews, or other forms without detailed empirical data.

**Figure 1:**
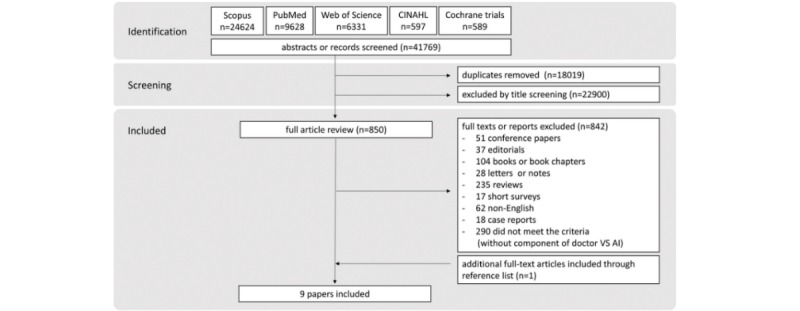
Flow diagram of study inclusion and exclusion process.

On the basis of the above inclusion and exclusion criteria, 2 reviewers (JS and BJ) independently screened article titles and abstracts and identified eligible articles. The full text of eligible articles was retrieved via the institutional access. Any discrepancy occurred during this process was resolved by discussion with 2 senior authors (WKM and CJPZ). The process of systematic search and the identification of reviewed articles are depicted in Figure 1.

### Data Extraction, Data Synthesis, and Quality Assessment

Characteristics of included studies were extracted independently by 2 reviewers (JS and BJ) after verification by 2 senior authors (WKM and CJPZ). The characteristics comprised (1) first author and publication year, (2) AI technology, (3) classification and labeling, (4) data sources (including the sample size of total sets, training sets, validation, and/or tuning sets and test sets), (5) training process, (6) internal validation methods, (7) human clinician reference, and (8) performance assessment.

Study quality was assessed using the Cochrane’s risk-of-bias tool [10]. This tool provides a domain-based approach to help reviewers judge the reporting of various types of risk by scrutinizing information from reviewed articles, and in turn, the judgment can be made based on these pieces of supporting information against specific types of risk of interest. The types of risk assessed in this review include (1) blinding of participants and personnel (performance bias), (2) blinding of outcome assessment (detection bias), (3) incomplete outcome data (attrition bias), and (4) selective reporting (reporting bias).

## Results

### Systematic Search

Following the systematic search process, 41,769 citations were retrieved from the database and 22,900 articles were excluded based on their titles and abstracts, resulting in 850 articles to be reviewed in detail. In addition, 842 articles were further excluded based on their full text. One article was identified from the manual searches. Finally, 9 studies were included for review (Figure 1).

### Characteristics of Included Studies

Table 1 summarizes the characteristics of these 9 studies. These 9 included studies were published between 2017 and 2019 and conducted across countries, including China, Germany, South Korea, the United Kingdom, and the United States. Regarding their studied medical conditions, 3 studies could be categorized under ophthalmology, including diabetic retinopathy [11], macular degeneration [11], and congenital cataracts [12], whereas another 3 studies focused on onychomycosis [13] and skin lesions/cancers [14,15]. The other studies related to radiology were focused on thoracic [16,17] and neurological [18] conditions.

A convolutional neural network (CNN) was the commonly applied advanced AI technology in all reviewed studies, with the exception of 1 study: González-Castro et al adopted support vector machine classifiers in their study [18].

Owing to the difference in study objectives, methodology, and medical fields, classification type between individual studies differed correspondingly. For instance, studies related to ophthalmological images [11,12] had differences in image sources (eg, ocular images [12] or optical coherence tomography [OCT]–derived images [11]) and, thus, the classification differed correspondingly (Table 1). Another study that was also based on OCT-derived images [19] focused on the referral suggestion made between clinical experts and AI, and the classification of multiple suggestion decisions was used. With regard to onychomycosis images, 4 and 6 classes were both used for training, and binary classification was subsequently used in testing by Han et al [13].

**Table 1:** Characteristics of included studies.

Authors (year)	Artificial intelligence technology	Classification/labeling	Data source; sample size of total dataset, training sets, validation and/or tuning sets and test-set	Training process	Internal validation	Human clinicians (external validation)
Brinker (2019) [14]	A convolutional neural network; CNN (trained with enhanced techniques on dermoscopic images)	All melanomas were verified by histopathological evaluation of biopsies; the nevi were declared as benign via expert consensus	International Skin Imaging Collaboration (ISIC) image archive; *Total:* 13,737; *Training:* 12,378 (1888 melanomas and 10,490 atypical nevi); *Validation:* 1359 (230 melanomas and 1129 atypical nevi); *Test:* 100 dermoscopic images	A ResNet50 CNN model (residual learning) used for the classification of melanomas and atypical nevi.	Not reported	One hundred and forty-five dermatologists from 12 German university hospitals (using 100 images)
De Fauw (2018) [19]	A segmentation CNN model using a 3-dimensional U-Net architecture	Referral suggestion: urgent/semi-urgent/routine/observation only (golden standard labels were retrospectively obtained by examining the patient clinical records to determine the final diagnosis and optimal referral pathway in the light of the subsequently obtained information)	Clinical OCT scans from Topcon 3D OCT, Topcon, Japan; *Device type 1: Training:* segmentation network*:* 877 (segmentation); gold standard referral decision: 14,884 (classification); *Validation:* 224 (segmentation); 993 (classification); *Test:* 997; *Device type 2: Training:* segmentation network: Additional 152 with 877 scans from device type 1 (segmentation); gold standard referral decision: 0 with 14,884 from device type 1 (referral decision); Validation: 112 (classification); *Test:* 116	1) Deep segmentation network, trained with manually segmented OCT scans; 2) Resulting tissue segmentation map; 3) Deep classification network, trained with tissue maps with confirmed diagnoses and optimal referral decisions; 4) Predicted diagnosis probabilities and referral suggestions.	Manually segmented and graded by 3 trained ophthalmologists, reviewed and edited by a senior ophthalmologist	*Device type 1:* 8 clinical experts (4 consultant ophthalmologists/retinal specialists and 4 optometrists trained in OCT interpretation and retinal disease); *Device type 2:* Five consultant ophthalmologists (4 of them were participants in the device type 1 and the other was new participant)
Esteva (2017) [15]	Deep CNNs (a GoogleNet Inception v3 CNN architecture pretrained on the ImageNet dataset)	Biopsy-proven clinical images with 2 critical binary classification, labeled by dermatologists	Eighteen different clinician-curated, open-access online repositories and clinical data from Stanford University Medical Center; *Total:* 129,405; *Training and validation:* 127,463 (9-fold cross validation); *Test:* 1942	1) Classification of skin lesions using a single CNN; 2) Trained end-to-end from images directly, using only pixels and disease labels as inputs	Two dermatologists (at both 3-class and 9-class disease partitions) using 9-fold cross-validation	Twenty-one board-certified dermatologists on epidermal and melanocytic lesion classification
Han (2018) [13]	A region-based convolutional deep neural network (R-CNN)	Four classes (onychomycosis, nail dystrophy, onycholysis, and melanonychia) and 6 classes (onychomycosis, nail dystrophy, onycholysis, melanonychia, normal, and others), manually categorized by dermatologists	Four hospitals (Asan Medical Center, Inje University, Hallym University, and Seoul National University); *Total:* 57,983; *Training:* 53,308 consist of datasets A1 (49,567) and A2 (3741); *Test:* 1358 consist of datasets B1 (100), B2 (194), C (125), and D (939)	1) Extracted clinical photographs automatically cropped by the R-CNN; 2) One dermatologist cropped all of the images from the A2, R-CNN model trained using information about the crop location; 3) fine image selector trained to exclude unfocused photographs; (4)Three dermatologists tagged clinical diagnosis to the nail images generated by the R-CNN, with reference to the existing diagnosis tagged in the original image; (5) ensemble model as the output of both the ResNet-152 and VGG-19 systems computed with the feedforward neural networks	Two classes (onychomycosis or not)	1) Forty-two dermatologists (16 professors, 13 clinicians with more than 10 years of experience in the department of Dermatology, and 8 residents) and 57 individuals from the general populations (11 general practitioners, 13 medical students, 15 nurses in the dermatology department, and 18 nonmedical persons) in the combined B1+C dataset; 2) The best 5 dermatologists among them in the combined B2+D dataset.
Kermany (2018) [11]	Deep CNN (also used transfer learning)	Four categories (3 labels): choroidal neovascularization or diabetic macular edema (labeled as *urgent referrals*), drusen (*routine referrals*), normal (*observation*); Binary classification also implemented (normal vs choroidal neovascularization/diabetic macular edema/drusen)	Optical coherence tomography (OCT) images selected from retrospective cohorts of adult patients from the Shiley Eye Institute of the University of California San Diego, the California Retinal Research Foundation, Medical Center Ophthalmology Associates, the Shanghai First People’s Hospital, and Beijing Tongren Eye Center between July 1, 2013 and March 1, 2017. *Total:* 207,130; *Training:* 108,312 (passed initial image quality review); *Validation:* 1000 (randomly selected from the same patients); *Test:* 1000 (independent sample from other patients)	After 100 epochs (iterations through the entire dataset), the training was stopped because of the absence of further improvement in both accuracy and cross-entropy loss	1000 images randomly selected from the images used for training (limited model)	Six experts with significant clinical experience in an academic ophthalmology center
Long (2017) [12]	Deep CNN	Binary classification by an expert panel in terms of opacity area (extensive vs limited), opacity density (dense vs nondense), and opacity location (central vs peripheral)	Childhood Cataract Program of the Chinese Ministry of Health (CCPMOH); *Total:* 1239; *Training:* 886; *Validation:* 5-fold cross validation for in silico test; 57 for multi-ospaital clinical trial; 53 for Web sited–based study; 303 for further validation; *Test:* 50	The championship model from the ImageNet Large Scale Visual Recognition Challenge 2014, containing 5 convolutional or down-sample layers in addition to 3 fully connected layers	K-fold cross-validation (K=5)	Three ophthalmologists with varying expertise (expert, competent, and novice)
Nam (2018) [16]	Deep learning–based automatic detection algorithm (DLAD)	Binary classification: normal or nodule chest radiographs (image-level labeling); Nodule chest radiographs were obtained from patients with malignant pulmonary nodules proven at pathologic analysis and normal chest radiographs on the basis of their radiology reports. All chest radiographs were carefully reviewed by thoracic radiologists.	Normal and nodule chest radiographs from three Korean hospitals (Seoul National University Hospital; Boramae Hospital; and National Cancer Center) and 1 US hospital (University of California San Francisco Medical Center). *Total:* 43,292; *Training:* 42,092 (33,467 normal and 8625 nodule chest radiographs); *Tuning:* 600 (300 normal and 300 nodule chest radiographs); *Internal validation:* 600 (300 normal and 300 nodule chest radiographs); *External validation/test:* 693	DLAD was trained in a semisupervised manner by using all of the image-level labels and partially annotated by 13 board-certified radiologists, with 25 layers and 8 residual connections	Radiograph classification and nodule detection performances of DLAD were validated by using 1 internal and 4 external datasets in terms of the area under ROC (AUROC) and figure of merit (FOM) form jackknife alternative free-response ROC (JAFROC)	18 physicians (including 3 nonradiology physicians, 6 radiology residents, 5 board-certified radiologists, and 4 subspecialty trained thoracic radiologists)
Rajpurkar (2018) [17]	Deep CNN with a 121-layer DenseNet architecture (CheXNeXt)	Binary values (absence/presence) in 14 pathologies: atelectasis, cardiomegaly, consolidation, edema, effusion, emphysema, fibrosis, hernia; Infiltration, mass; nodule, pleural thickening, pneumonia, and pneumothorax, obtained using automatic extraction methods on radiology reports	ChestX-ray14 dataset; *Total:* 112,120; *Training:* 98,637; *Tuning*: 6351; *Validation:* 420	1) Multiple networks were trained on the training set to predict the probability that each of the 14 pathologies is present in the image; 2) A subset of those networks, each chosen based on the average error on the tuning set, constituted an ensemble that produced predictions by computing the mean over the predictions of each individual network	Comprehensive comparison of the CheXNeXt algorithm to practicing radiologists across 7 performance metrics (ie, no external validation)	Nine radiologists (6 board-certified radiologists and 3 senior radiology residents from 3 institutions)
González-Castro (2017) [18]	Support vector machine (SVM) classifier	Binary classifier of the burden of enlarged perivascular spaces (PVS) as low or high	Data from 264 patients in Royal Hallamshire Hospital; *Total:* 264 (randomly partitioned into 5 equal-sized subsets); *Training:* 4 of the 5 subsets (~211); *Test:* one of the five subsets (~53)	Several combinations of the regularization parameter C and gamma, were used and assessed with all descriptors to find the optimal configuration using the implementation provided by the libSVM library	A stratified 5-fold cross-validation repeating ten times	Two observers (an experienced neuroradiologist and a trained image analyst)

Similarly, the training processes employed in individual studies were not identical to each other because of their field-specific nature and classification-peculiar algorithms. For instance, predictions in 1 ophthalmological study [11] were informed by a model using transfer learning on a Web-based platform on the basis of training on graded OCT images. The other ophthalmological study [12] focusing on congenital cataracts employed a 3-stage training procedure (ie, identification, evaluation, and strategist networks) to establish a collaborative disease management system beyond only disease identification. Owing to this, data sources for training were field specific. The training procedures in the other studies are detailed in Table 1.

Furthermore, 2 studies [11,16] employed both internal and external validation methods via training and/or validating the effectiveness of their AI algorithms using images from their own datasets and external datasets. Kermany et al investigated the effectiveness of their AI systems in the prediction of a diagnosis in their own ophthalmological images as well as the generalizability to chest x-ray images [11]. In contrast, Nam et al validated their work using datasets from not only their own hospital but also other different local or overseas hospitals [16]. The remaining studies did not report both internal or external validation or differentiate either.

Variation in dataset size was also observed. Specifically, the quantity of training sets, validation (and tuning) sets, and test sets ranged from 211 to approximately 113,300, from 53 to approximately 14,163, and from 50 to 1942, respectively.

### Performance Indices and Comparison Between Artificial Intelligence and Clinicians

All studies compared the diagnostic performance between AI and licensed doctors (see Table 2). Performance indices used for comparison included diagnostic accuracy, weighted errors, sensitivity, specificity (and/or the area under the receiver operating characteristic curve [AUC]), and false-positive rate. A total of 4 articles [11,12,15,17] adopted the *accuracy* (ie, the proportion of true results [both positives and negatives] among the total number of cases examined) to compare diagnostic performance between AI and humans. Long et al observed a high accuracy in AI (90%-100%) compared with a panel of specialty doctors’ predefined diagnostic decision and transcended the average levels of clinicians in most clinical situations except for treatment suggestion. Esteva et al also found that AI achieved comparable accuracy with or outperformed their human rivals (AI vs dermatologists: 72.1% (SD 0.9%) vs 65.8% using 3-class disease partition and 55.4% (SD 1.7%) vs 54.2% using 9-class disease partition [15]). The same was also observed in the study by Rajpurkar et al [17], indicating an agreement in results between AI and radiologists. Similarly, Kermany et al showed that their AI achieved high accuracy (96.6%) while acknowledging that their 6 experienced ophthalmologists still performed well [11]. They also reported weighted errors in which medical doctors maintained better accuracy (4.8% vs 6.6%). De Fauw et al [19] reported unweighted errors by using 2 devices, and the results showed their AI’s performance commensurate with retina specialists and generalizable to another OCT device type.

Overall, 7 studies [11,13-18] compared the sensitivity, specificity, and/or AUC between AI and medical experts. Overall, the performance of the algorithm was on par with that in human experts and significantly superior to those experts with less experience [11,13,16,18] (Table 2).

False-positive rates between AI and clinicians were compared in 2 studies [12,16]. The number of false discoveries occurring in AI was approximate to that by expert and competent ophthalmologists with respect to image evaluation (AI vs expert or competent: 9 vs 5 or 11) and treatment suggestion (AI vs expert or competent: 5 vs 1 or 3) but was lower than that of novice ophthalmologists with 5 versus 12 and 8, regarding image evaluation and treatment suggestion, respectively [12]. The other study also found the false-positive rate of their deep learning algorithm in nodule detection being close to the average level of thoracic radiologists (0.3 vs 0.25) [16].

Other performance indices were compared in single studies. Apart from false positives, Long et al also compared the number of missed detections between their AI and ophthalmologists, and their AI outperformed (ie, fewer missed detections) all ophthalmologists with varying expertise (expert, competent, and novice). The time to interpret the tested images between AI and human radiologists was reported by Rajpurkar et al [16] The authors also compared AI and radiologists with respect to positive and negative predictive values, Cohen kappa, and F1 metrics (Table 2).

**Table 2:** Comparison between artificial intelligence and human clinicians.

Authors (year)	Performance index (AI^a^ vs human clinicians)						
	Accuracy	AUC^b^	Sensitivity	Specificity	Error/weighted error	False positives	Other indices
Brinker (2019) [14]	N/A^c^	Details provided in the article	Sensitivity (at specificity=73.3%):86.1% ; versus ;86.7% (among 3 resident dermatologists)	Specificity (at sensitivity=89.4%): mean=68.2% (range: 47.5%-86.25%) versus mean=64.4% (all 145 dermatologists, range: 22.5%-92.5%); Specificity (at sensitivity=92.8%): mean=61.1% versus mean=57.7 % (among 16 attending dermatologists)	N/A	N/A	N/A
De Fauw (2018) [19]	N/A	No comparison	N/A	N/A	*Device type 1:* Error rate: 5.5% versus 2 best retina specialists: 6.7% and 6.8% (performed comparably with 2 best and significantly outperformed the other 6 experts); *Device type 2:* Error rate: 3.4% versus 2.4% (average) (Details provided in the article)	N/A	N/A
Esteva (2017) [15]	*(Internal validation with 2 dermatologists);Three-class disease partition:* 72.1% (SD 0.9%) versus 65.56% and 66.0%; *Nine-class disease partition:* 55.4% (SD1.7) versus 53.3% and 55.0%	AUC of AI was reported but no comparison with human clinicians (Details provided in the article)	AI outperformed the average of dermatologists; (Details provided in the article)	AI outperformed the average of dermatologists (Details provided in the article)	N/A	N/A	N/A
González-Castro (2017) [18]	N/A	AUC (model 1): 0.9265 versus 0.9813 and 0.9074; AUC (model 2): 0.9041 versus 0.8395 and 0.8622; AUC (model 3): 0.9152 versus 0.9411 and 0.8934	N/A	N/A	N/A	N/A	N/A
Han (2018) [13]	N/A	N/A	Youden index (sensitivity + specificity - 1): B1+C dataset: >67.62% (trained with A1 dataset) and >63.03% (trained with A2 dataset) vs 48.39% (99% CI 29.16% (SD 67.62%); 95% CI 33.76% (SD 63.03%); B2+D dataset: Only one dermatologist performed better than the ensemble model trained with the A1 dataset, and only once in three experiments		N/A	N/A	N/A
Kermany (2018) [11]	96.6% versus 95.9% (mean; range: 92.1%-99.7%)	N/A	97.8% versus 99.3% (mean; range: 98.2%-100%)	97.4% versus 95.4% (mean; range: 82%-99.8%)	6.6% versus 4.8% (mean; range: 0.4%-10.5%)	N/A	N/A
Long (2017) [12]	Accuracy (distinguishing patients and healthy individuals): 100% versus 98% (expert), 98% (Competent), 96% (novice) [mean=97.33%]; *Accuracy (opacity areas):* 90% versus 90% (expert), 84% (competent), 78% (novice) [mean=84%]*Accuracy (densities):* 90% versus 90% (expert), 90% (competent), 86% (novice) [mean=88.7%]; *Accuracy (location):* 96% versus 88% (expert), 88% (competent), 86% (novice) [mean=82.7%]; Accuracy (treatment suggestion): 90% versus 92% (expert), 92% (competent), 82% (novice) [mean=88.7%]	N/A	N/A	N/A	N/A	*Number of false positive in 50 cases;* Evaluation network (opacity area, density and location): 9 versus 5 (expert), 11 (competent), 12 (novice); Strategist network (treatment suggestion): 5 versus 1 (expert), 3 (competent), 8 (novice)	*Missed detections:* Evaluation network (opacity area, density and location): 4 versus 11 (expert), 8 (competent), 20 (novice) Strategist network (treatment suggestion): 0 versus 3 (expert), 1 (competent), 1 (novice)
Nam (2018) [16]	N/A	AUROC (in radiograph classification): 0.91 versus mean=0.885 (DLAD higher than 16 physicians and significantly higher than 11); JAFROC FOM (in nodule detection): 0.885 versus mean=0.794 (DLAD higher than all physicians and significantly higher in 15)	80.7% versus mean=70.4%	No report of physicians’ performance	N/A	0.3 versus mean=0.25	N/A
Rajpurkar (2018) [16]	Mean proportion correct value for all pathologies: 0.828 (SD=0.12) versus 0.675 (SD=0.15; board-certified radiologists) and 0.654 (SD=0.16; residents)	AUC (cardiomegaly): 0.831 versus 0.888 (*P*<.05); AUC (emphysema): 0.704 versus 0.911 (*P*<.05); AUC (hernia): 0.851 versus 0.985; (*P*<.05); AUC (atelectasis): 0.862 versus 0.808 (*P*<.05); No significant difference for other 10 pathologies	CheXNEXt versus board-certfied radiologists *only;* Sensitivity (masses): 0.754 (95% CI 0.644-0.860) versus 0.495 (95% CI 0.443-0.546); Sensitivity (nodules): 0.690 (95% CI 0.581-0.797) vs 0.573 (95% CI 0.525-0.619); Sensitivity (consolidation): 0.594 (95% CI 0.500-0.688) versus 0.456 (95% CI 0.418-0.495); Sensitivity (effusion); 0.674 (95% CI 0.592-0.754) versus 0.761 (95% CI 0.731-0.790); (detailed comparison on other 10 pathologies are available in the original article)	CheXNEXt versus board-certfied radiologists *only*; Specificity (masses): 0.911 (95% CI 0.880-0.939) versus 0.933 (95% CI 0.922-0.944); Specificity (nodules): 0.900 (95% CI 0.867-0.931) versus 0.937 (95% CI 0.927-0.947) Specificity (consolidation): 0.927 (95% CI 0.897-0.954) versus 0.935 (95% CI 0.924-0.946) Specificity (effusion); 0.921 (95% CI 0.889-0.951) versus 0.883 (95% CI 0.868-0.898); (detailed comparison on other 10 pathologies are available in the original article)	N/A	N/A	*Time to interpret the 420 images:* 1.5 min versus 240 min (range 180-300 min); *Positive and negative predictive values; Cohen’s kappa F1 metric* **(**Details provided in the Appendices of the article)

^a^AI: artificial intelligence.

^b^AUC: area under the receiver operating characteristic curve.

^c^Not applicable.

### Quality Assessment of Included Studies

**Figure 2:**
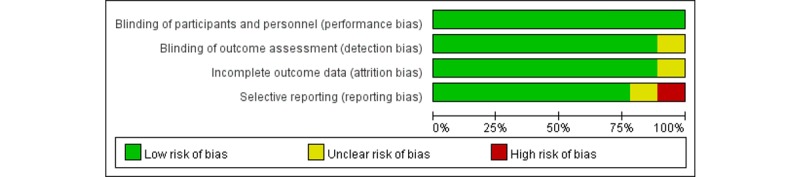
Distribution of bias in the included studies.

**Figure 3:**
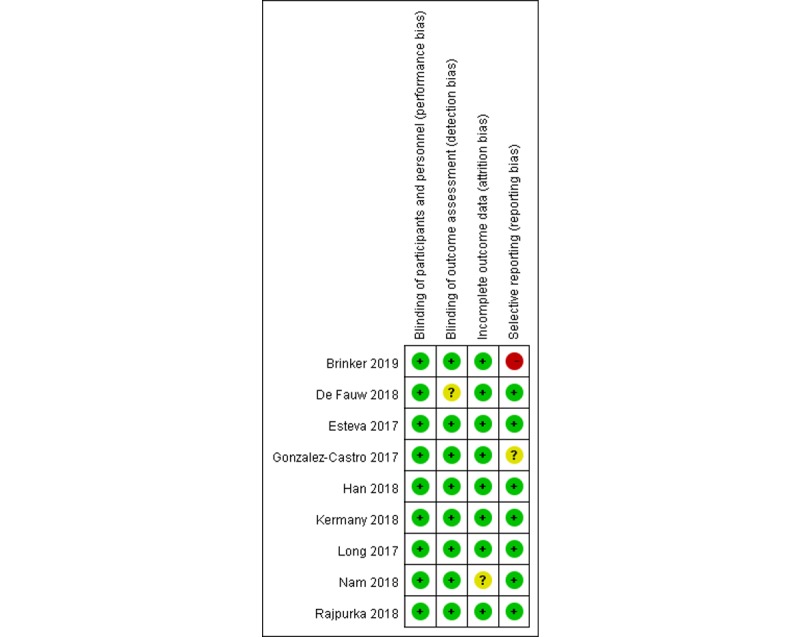
Risk of bias in the included studies.

The methodological quality of included studies (see Figures 2 and 3) was assessed using the Cochrane’s risk-of-bias tool [10]. This tool was designed to assist the assessment on the risk of bias in reviewed articles based on their reporting in terms of specified domains. The evaluation is grounded on whether individual articles provided supporting details, and the summary is presented as high, low, or unclear bias in graphs. Overall, most of reviewed studies had a low risk of bias with respect to the specified domains (Figures 2 and 3). A total of 3 studies were classified as *unclear risk* in particular domains. Specifically, there was no report on whether blinding of participants and personnel (related to performance bias) was observed in the study by De Fauw et al [19]. The study by González-Castro et al [18] was classified as unclear risk in terms of selective reporting (reporting bias) because of failing to report all prespecified performance indices. Attrition bias rising from incomplete outcome data (ie, physicians’ performance) was not assessable based on the reporting by Nam et al [16] (see Multimedia Appendix 2 for details).

## Discussion

### Principal Findings

Our systematic review identified 9 articles on advanced AI applications for disease diagnosis. These spanned multiple medical subjects, including retinal diseases, skin cancers, pulmonary nodules, and brain tumors. Although several articles covered similar medical topics, distinct AI algorithms and training processes were employed across articles. The validation methods of AI algorithm effectiveness also varied between articles. According to our inclusion criteria, only articles encompassing comparisons of diagnostic performance between advanced AI and clinical experts were reviewed.

The literature has shown that AI has comparable performance with medical experts. Major advanced AI approaches such as deep learning and CNNs yield significant discriminative performance upon provision of sufficient training datasets. In addition to relatively high sensitivity and specificity in object-identifying tasks [11,15], the advantages of AI have also been visible in the instantaneity of reporting and consistency of producing results [17]. Although neural network approaches generally require substantial data for training, recent research suggested that it may be feasible to apply AI to rare diseases [11,12] and, in particular circumstances, to databases where a large number of examples are not available. The combination with other technologies such as a cloud-based data-sharing platform would extend AI’s likely use beyond clinical settings or spatial limits [20].

Most AI achievements can be observed in image recognition [21]. Object-identification tasks were the main applications in medical diagnoses across the reviewed articles. Computer-assisted technologies facilitate the rapid detection of clinical symptoms of interest (eg, benign and malignant) based on image features (eg, tone and rim) resulting in consistent outputs. AI-based classification of physical characteristics via vast numbers of examples is reinforced during training, and this ability is consolidated and gradually levels the discriminative academic performance in appearance-based diagnoses such as skin diseases [15,21]. Such AI-assisted imaging-related clinical tasks can reduce the cognitive burden on human experts [17] and thus increase the efficiency of health care delivery.

AI performs at par with human experts in terms of image analysis. Image analysis involves a number of object-identification tasks whose outputs rely exclusively on the detection and interpretation of concrete features such as shapes and colors. The nonfatigue characteristic of advanced artificial networking enables constant training and learning until achieving satisfactory accuracy [17]. This shows marked success in disease diagnoses related to image evaluation. This unique advantage of AI, which humans are biologically unlikely to possess, contributed to its performance exceeding that of clinical professionals, as seen in the reviewed articles.

The literature shows that almost every achievement of AI is established based on diagnosis outcomes. However, any assessment of diagnostic outcomes needs to yield meaningful implications. The diagnostic criteria are developed based on long-standing and recursive processes inclusive of real-world practice appraised by clinicians, as summarized in Table 1. Although the recently promising self-learning abilities of AI may lead to additional prospects [22], the viability of such diagnostic processes is inevitably determined by human experts through cumulative clinical experience [23,24]. In other words, clinical experts are the go-to persons informing AI of what the desired predictions are. AI is still incapable of interpreting what it has obtained from data and of providing telling results. Therefore, the final success of AI is conditionally restricted by medical professionals who are the real evaluators of their diagnostic performance. This signifies its *artificial* nature in a human-dominated medical environment.

Given such a relationship between AI and human users, the applicability of advanced AI and clinical significance cannot be isolated. The development of AI technology itself may provide an encouraging outlook on medicine applications, but an evaluation conducted by medical specialists plays a fundamental role in AI’s continued blooming. In medical applications, AI cannot exist without human engagement because the final diagnoses need to have real-world implications. Patient-oriented medicines specify the essence of patient data in the AI establishment and learning process. Each successful AI, regardless of whether it is database driven or self-learning, needs to eventually improve patients’ health. The tireless learning abilities of AI can complement cognitive fatigue in humans [17] and can substantially improve clinical efficiency. Its outstanding performance, comparable with that of experts, saves huge amounts of time in clinical practice, which, in turn, alleviates the tension in the long-established process of the transition from novice clinician to expert.

Despite being a propitious moment for AI, there are issues to be addressed in the coming stages. It remains unclear whether AI can transform the current clinician-dominant assessment in clinical procedures. It is not surprising that a hybrid system contributed by both AI and physicians would produce more effective diagnostic practices, as evidenced by 1 of the reviewed articles [17]. This could, in turn, bring about improved health care. Data interpretation still appears to be a significant challenge to AI. Future research may focus more on this topic.

### Comparison With Previous Work

Before this review, several reviews on general AI application have been available in the specific fields such as neurosurgery, digital dermoscopy, and interpretation of intrapartum fetal heart rate [25-27]. However, most of these reviews did not limit their scope to advanced AI or deep learning, which is deemed to be an emerging interest to health care professionals in terms of disease diagnoses. Our review particularly compared the diagnostic performance of advanced AI with that of clinician experts, providing an updated summary on latest development of AI applications to disease diagnoses. Our findings suggest that AI’s diagnostic performance is at par with clinical experts, and the streamlined efficiency of AI transcends human doctors. Acknowledging the practical value of AI added to current practice, the underpinning of human clinical experience and patient-centered principle should remain in the future AI application to disease diagnoses.

### Limitations

Our review systematically searched articles published in selected major databases. According to our preset inclusion and exclusion criteria, we did not specifically review the conference abstracts that may contain the most developed AI that can inform diagnostic practice. Only English articles were included in this review, and thus relevant studies published in other languages may have been missed.

### Conclusions

In summary, current AI developments have achieved comparable performance with medical experts in specific fields. Their predictive performance and streamlined efficiency pertaining to disease diagnoses—particularly in medical imaging tasks—have transcended that of clinicians because of their tireless and stable characteristics. Further studies can be focused on other medical imaging such as magnetic resonance imaging and other image-unrelated medical practices [28,29]. With the continued development of AI-assisted technologies, the clinical implications underpinned by clinicians’ experience and guided by patient-centered health care principles should be considered in future AI-related and technology-based medical research.

## Appendix

Multimedia Appendix 1 Search terms used to identify articles related to telemedicine and related technology used in disease diagnoses.

Multimedia Appendix 2 Risk of bias table for individual studies.

## Abbreviations

AI: artificial intelligence
AUC: area under the receiver operating characteristic curve
CNN: convolutional neural network
OCT: optical coherence tomography
