# Supplementary material for: Artificial Intelligence Versus Clinicians in Disease Diagnosis: Systematic Review
Source: JMIR Med Inform. 2019 Aug 16;7(3):e10010. doi: 10.2196/10010 (PMC6716335; doi:10.2196/10010)
Supplement: Multimedia Appendix 1 [file medinform_v7i3e10010_app1.pdf]

Textbox 1. Search terms used to identify articles related to telemedicine and related technology used in disease diagnoses.

1. artificial intelligen*
2. computational intelligen*
3. machine intelligen*
4. deep learning
5. deep neural network
6. machine learning
7. 1 or 2 or 3 or 4 or 5 or 6
8. medic*
9. surgery
10. surgical
11. cancer
12. diabetes
13. diabetic
14. dermatologist
15. photographs
16. microscopy
17. specimen
18. nodule*
19. 8 or 9 or 10 or 11 or 12 or 13 or 14 or 15 or 16 or 17 or 18
20. diagnos*
21. prediction
22. detection
23. classification
24. 20 or 21 or 22 or 23
25. 7 and 19 and 24
