# Supplementary material for: Artificial Intelligence Versus Clinicians in Disease Diagnosis: Systematic Review
Source: JMIR Med Inform. 2019 Aug 16;7(3):e10010. doi: 10.2196/10010 (PMC6716335; doi:10.2196/10010)
Supplement: Multimedia Appendix 2 [file medinform_v7i3e10010_app2.pdf]

Supplementary Table 1. Risk of bias table for individual studies

Brinker（2019）

Entry	Judgement	Support for judgement
random sequence generation (selection bias)	low risk	quote:" The training and validation images were also selected using a random generator from the set of available images in the ISIC archive, excluding the already selected test images."<br>-describe a random component
allocation concealment (selection bias)	low risk	quote: “The training and validation images were also selected using a random generator from the set of available images in the ISIC archive, excluding the already selected test images."<br>- participants and investigators could not foresee assignment because a method used to conceal allocation
blinding of participants and personnel (performance bias)	low risk	quote: "all the dermatologists voluntarily participating in the reader study were anonymous"<br>"training of an artificial intelligence algorithm was conducted with open source images"<br>- Blinding of participants and key study personnel ensured, and unlikely that the blinding could have been broken.
blinding of outcome assessment (detection bias)	low risk	quote: “The first part of both questionnaires was identical and recorded age, sex, years of dermatologic practice/experience, estimated number of skin checks performed and position within the medical hierarchy. This was followed by 100 dermoscopic (link 1) or clinical (link 2) images of 80 benign nevi and 20 biopsy-verified melanomas, each.”<br>-Blinding of outcome assessment ensured, and unlikely that the blinding could have been broken.
incomplete outcome data (attrition bias)	low risk	No missing outcome data
selective reporting (reporting bias)	high risk	did not report AUC of algorithms<br>- Not all of the study’s pre-specified primary outcomes have been reported.

De Fauw (2018)

Entry	Judgement	Support for judgement
random sequence generation (selection bias)	low risk	quote: “The dataset selection and stratification process is displayed in a CONSORT flow diagram."<br>"all scans were read in a random order twice with at least a week between readings."<br>-describe a random component
allocation concealment (selection bias)	low risk	quote: “The dataset selection and stratification process is displayed in a CONSORT flow diagram in Supplementary Fig. 11."<br>- participants and investigators could not foresee assignment because a method used to conceal allocation
blinding of participants and personnel (performance bias)	low risk	quote: "Junior and senior graders were separate to those participating in the evaluation of expert performance.” "Only de-identified retrospective data was used for research, without the active involvement of patients.”<br>-Blinding of participants and key study personnel ensured, and unlikely that the blinding could have been broken.
blinding of outcome assessment (detection bias)	unclear	Quote: "Participants included four consultant ophthalmologists at Moorfields Eye Hospital with fellowship-level subspecialty training in medical retinal disease and extensive clinical experience (21, 21, 12.5 and 11.5 years of experience respectively), and four optometrists at Moorfields Eye Hospital with specialist training in OCT interpretation and retinal diseases (15, 9, 6 and 2.5 years of experience respectively). These are referred to as retinal specialists 1 to 4 and optometrists 1 to 4 in the rest of the paper (Supplementary Table 10). Each expert was instructed to provide a triage decision (Supplementary Table 1) and to record the presence or absence of the defined pathological features (Supplementary Table 5)."<br>- Insufficient information to permit judgement of ‘low risk’ or ‘high risk’.
incomplete outcome data (attrition bias)	low risk	No missing outcome data
selective reporting (reporting bias)	low risk	validation reports include all expected outcomes.

Esteva (2017)

Entry	Judgement	Support for judgement
random sequence generation (selection bias)	low risk	quote: “Connected components of this graph were not allowed to straddle the train/validation split and were randomly assigned to either train or validation."<br>-using a computer random number generator
allocation concealment (selection bias)	low risk	quote: “The number of images used for Fig. 3b was based on the availability of biopsy labelled data (that is, malignant melanocytic lesions are exceedingly rare compared to benign melanocytic lesions).’<br>“These numbers are statistically justified by the standards of the ILSVRC computer vision challenge6, which has 50-100 images per class for validation and test sets.<br>"For Fig. 3a, 140 images were randomly selected from each set of Fig. 3b, and a non-tested dermatologist (blinded to diagnosis) removed any images of insufficient resolution.”<br>-central allocation web based
blinding of participants and personnel (performance bias)	low risk	quote: “non-tested dermatologist (blinded to diagnosis) removed any images of insufficient resolution."<br>- Blinding of participants and key study personnel ensured, and unlikely that the blinding could have been broken.
blinding of outcome assessment (detection bias)	low risk	Test sets are available. Only training/validation sets are conditionally available (subject to reasonable request).
incomplete outcome data (attrition bias)	low risk	all outcome data is reported in fig. 3<br>-no missing outcome data
selective reporting (reporting bias)	low risk	-validation reports include all expected outcomes.

Gonzalez-Castro (2017)

Entry	Judgement	Support for judgement
random sequence generation (selection bias)	low risk	Quote: “The whole set, represented by the descriptors explained in ‘Descriptors based on the Wavelet transform’, ‘Local binary patterns’ and ‘BoW’ sections, was randomly partitioned into five equal-sized subsets with the same distribution as the original set.”<br>-describe a random component
allocation concealment (selection bias)	unclear	the method of concealment is not described.
blinding of participants and personnel (performance bias)	low risk	quote: ” imaging confounds visually identified by Observer 2 in the BG region blind to the neuroradiological reports.”<br>-Blinding of participants and key study personnel ensured, and unlikely that the blinding could have been broken.
blinding of outcome assessment (detection bias)	low risk	"The ratings were done blind to all clinical information, each other’ s results and any intermediate or final computational results."<br>-Blinding of outcome assessment ensured, and unlikely that the blinding could have been broken.
incomplete outcome data (attrition bias)	low risk	-No missing outcome data.
selective reporting (reporting bias)	unclear risk	quote: "The overall results were validated in terms of accuracy, sensitivity and specificity, using the dichotomized ratings of Observer 1 as ground truth."<br>failed to report all accuracy, sensitivity and specificity of observers.<br>- they reported AUC which were calculated based on sensitivity and specificity, but no report on Accuracy.

Han (2018)

Entry	Judgement	Support for judgement
random sequence generation (selection bias)	unclear	quote: "We created dataset ªAº (Asan Medical Center) with 598,854 clinical images acquired from 2003 to 2016 and then used different methods to generate the A1 and A2 datasets."<br>"we used R-CNN to automatically identify and extract part of the nail from individual clinical images"<br>-insufficient information about the sequence generation process to permit judgement of 'low risk' or 'high risk'.
allocation concealment (selection bias)	low risk	quote:” CNN hand & foot image selector which automatically detects and selects hand and foot images from the entire dataset.”<br>” CNN fine image selector which rules out photographs with inadequate composition or focus.”<br>-computer based randomization method used to conceal allocation.
blinding of participants and personnel (performance bias)	low risk	quote: “Data on patient demographics and clinical images were collected via a retrospective chart review, and all data were fully anonymized before we accessed them.”<br>-Blinding of participants and key study personnel ensured, and unlikely that the blinding could have been broken.
blinding of outcome assessment (detection bias)	low risk	quote: “Data on patient demographics and clinical images were collected via a retrospective chart review, and all data were fully anonymized before we accessed them.”<br>-Blinding of outcome assessment ensured, and unlikely that the blinding could have been broken.
incomplete outcome data (attrition bias)	low risk	No missing outcome data
selective reporting (reporting bias)	low risk	validation reports include all expected outcomes.

Kermany (2018)

Entry	Judgement	Support for judgement
random sequence generation (selection bias)	low risk	quote: "The locally connected (convolutional) layers are frozen and transferred into a new network, while the final, fully connected layers are recreated and retrained from random initialization on top of the transferred layers."<br>quote: "Before training, each image went through a tiered grading system consisting of multiple layers of trained graders of increasing expertise for verification and correction of image labels. Each image imported into the database started with a label matching the most recent diagnosis of the patient."<br>-using a computer random number generator
allocation concealment (selection bias)	low risk	quote: “Workflow diagram showing overall experimental design describing the flow of optical coherence tomography (OCT) images through the labeling and grading process followed by creation of the transfer learning model, which then underwent training and subsequent testing.”<br>-central allocation web based
blinding of participants and personnel (performance bias)	low risk	quote: “The second tier of graders consisted of four ophthalmologists who independently graded each image that had passed the first tier. "<br>"To account for human error in grading, a validation subset of 993 scans was graded separately by two ophthalmologist graders, with disagreement in clinical labels arbitrated by a senior retinal specialist."<br>- Blinding of participants and key study personnel ensured, and unlikely that the blinding could have been broken.
blinding of outcome assessment (detection bias)	low risk	quote: "outcome evaluations AUC, errors, accuracy"<br>-Blinding of outcome assessment ensured, and unlikely that the blinding could have been broken.
incomplete outcome data (attrition bias)	low risk	-no missing outcome data
selective reporting (reporting bias)	low risk	plots showing binary performance in both training and validation datasets in fig S1. Receiver operating characteristic curves for binary classifiers in Fig S2.<br>-validation reports include all expected outcomes.

Long (2017)

Entry	Judgement	Support for judgement
random sequence generation (selection bias)	unclear	insufficient information about the sequence generation process to permit judgement of 'low risk' or /High risk'
allocation concealment (selection bias)	unclear	concealment is not described
blinding of participants and personnel (performance bias)	low risk	quote: "Each image was independently described and labelled by two experienced ophthalmologists, and a third ophthalmologist was consulted in the case of<br>disagreement. “<br>“Two of the authors (E.L. and H.L.) completed the searches independently.<br>"Expert panel Independently evaluates."<br>- Blinding of participants and key study personnel ensured, and unlikely that the blinding could have been broken.
blinding of outcome assessment (detection bias)	low risk	quote: "In this test, three ophthalmologists of varying expertise (expert, competent and novice) were asked to independently complete the same test paper as the intelligence agent without any additional information."<br>" The expert panel was blind and had no access to the deep-learning predictions."<br>"The deep-learning predictions were time-stamped, verified and saved by an individual who was blinded to the expert-panel labels to ensure that there was no information leak or double-dipping when comparing the predictions against expert panel labels."<br>-Blinding of outcome assessment ensured, and unlikely that the blinding could have been broken.
incomplete outcome data (attrition bias)	low risk	quote: “CC-Cruiser also provided accurate detections for all the normal and surgery cases.”<br>-No missing outcome data.
selective reporting (reporting bias)	low risk	-validation reports include all expected outcomes

Nam (2018)

Entry	Judgement	Support for judgement
random sequence generation<br>(selection bias)	low risk	quote: "Thereafter, the chest radiography data were randomly assigned into one of the following three data sets: The patients in the three data sets were different and exclusive to each of the other data sets."<br>-using a computer random number generator
allocation concealment<br>(selection bias)	unclear	quote: “chest radiography data randomly assigned”<br>-central allocation web based
blinding of participants and personnel<br>(performance bias)	low risk	quote: “chest radiographs labeled by radiologists blinded to other radiologists' labeling and annotation.”<br>-Blinding of participants and key study personnel ensured, and unlikely that the blinding could have been broken.
blinding of outcome assessment<br>(detection bias)	low risk	quote: “In test 1, each observer independently reviewed each chest radiograph to discriminate normal chest radiographs from nodule chest radiographs (radiograph classification) and localized lung nodules (nodule detection) on a five-point confidence scale without DLAD.”<br>-blinding of outcome assessment ensured, and unlikely that the blinding could have been broken.
incomplete outcome data (attrition bias)	unclear	Insufficient exclusions to permit judgement of ‘Low risk’ or ‘High risk’.
selective reporting (reporting bias)	low risk	-validation reports include all expected outcomes

Rajpurkar (2018)

Entry	Judgement	Support for judgement
random sequence generation (selection bias)	unclear	quote: "The dataset is currently the largest public repository of radiographs, containing 112,120 frontal-view (both posteroanterior and anteroposterior) chest radiographs of 30,805 unique patients."<br>-insufficient information about the sequence generation process to permit judgement of 'low risk' or 'high risk'
allocation concealment (selection bias)	low risk	quote: "a subset of those networks, each chosen based on the average error on the tuning set."<br>- participants and investigators could not foresee assignment because a method used to conceal allocation
blinding of participants and personnel (performance bias)	low risk	quote: "The dataset is currently the largest public repository of radiographs, containing 112,120 frontal-view (both posteroanterior and anteroposterior) chest radiographs of 30,805 unique patients."<br>"the parameters are automatically learned from a large amount of data labeled with the presence or absence of each pathology."<br>-Blinding of participants and key study personnel ensured, and unlikely that the blinding could have been broken.
blinding of outcome assessment (detection bias)	low risk	quote: "Radiologists did not have access to any patient information or knowledge of disease prevalence in the data."<br>-Blinding of outcome assessment ensured, and unlikely that the blinding could have been broken.
incomplete outcome data (attrition bias)	low risk	No missing outcome data
selective reporting (reporting bias)	low risk	validation reports include all expected outcomes.
